# Supplementary material for: Novel Somatic Genetic Variants as Predictors of Resistance to EGFR-Targeted Therapies in Metastatic Colorectal Cancer Patients
Source: Cancers (Basel). 2020 Aug 11;12(8):2245. doi: 10.3390/cancers12082245 (PMC7463997; doi:10.3390/cancers12082245)
Supplement: Supplementary file 1 [file cancers-12-02245-s001.pdf]

Supplementary figure S1.

a) Resistant patients

KRAS: NM_004985.4: c.34G>T; p.(G12C) (P11)

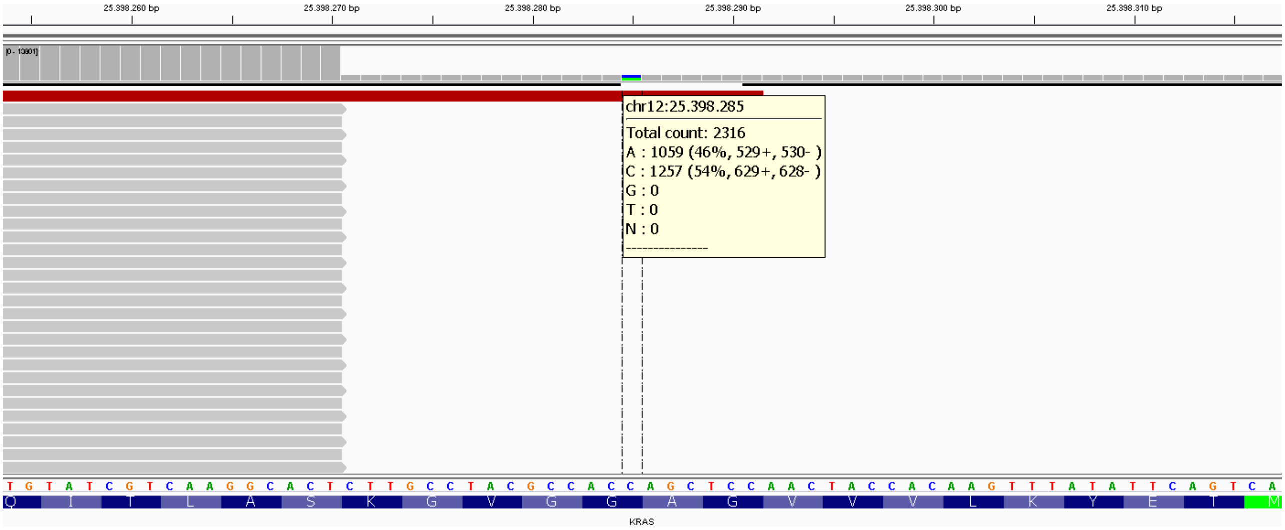

KRAS: NM_004985.4: c.182A>T; p.(Q61L) (P39)

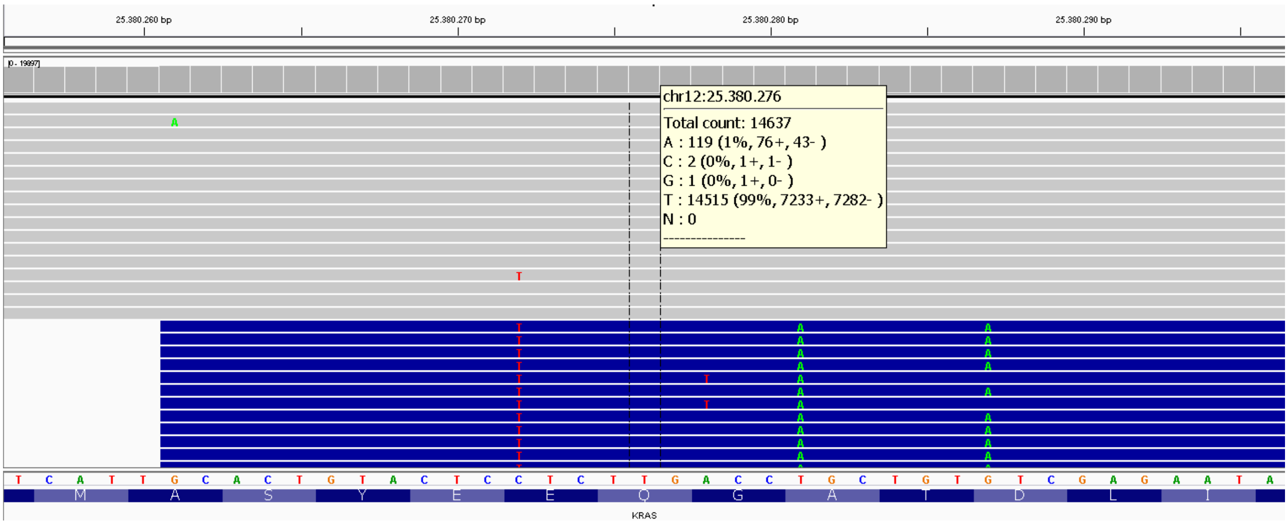

NRAS: NM_002524.4: c.34G>A; p.(G12S) (P51)

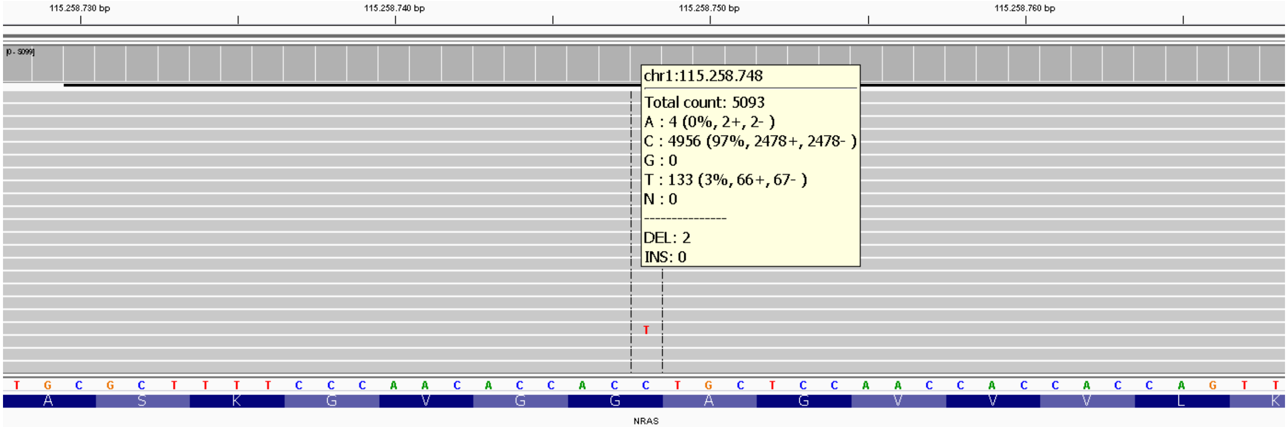

NRAS: NM_002524.4: c.38G>A; p.(G13D)

P55

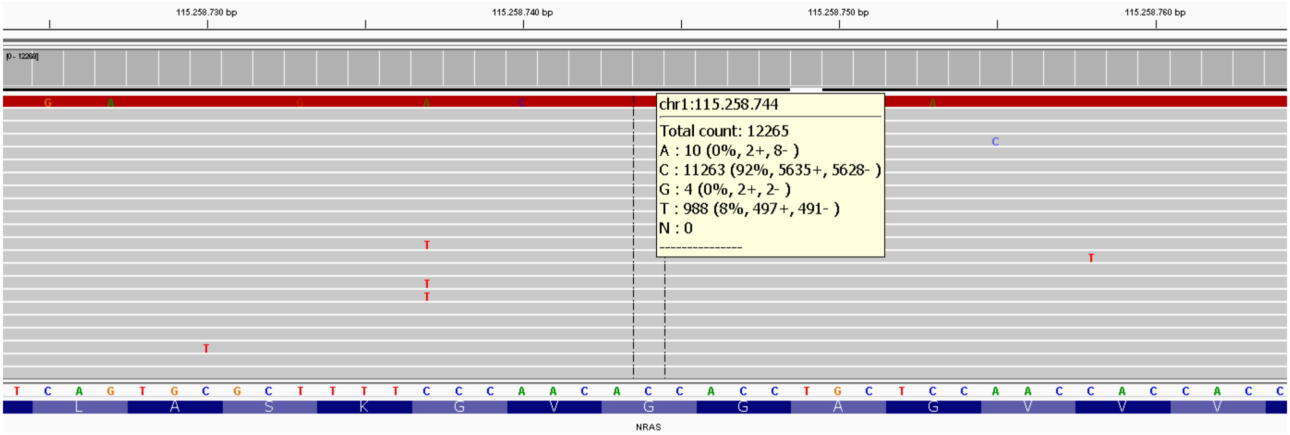

P57

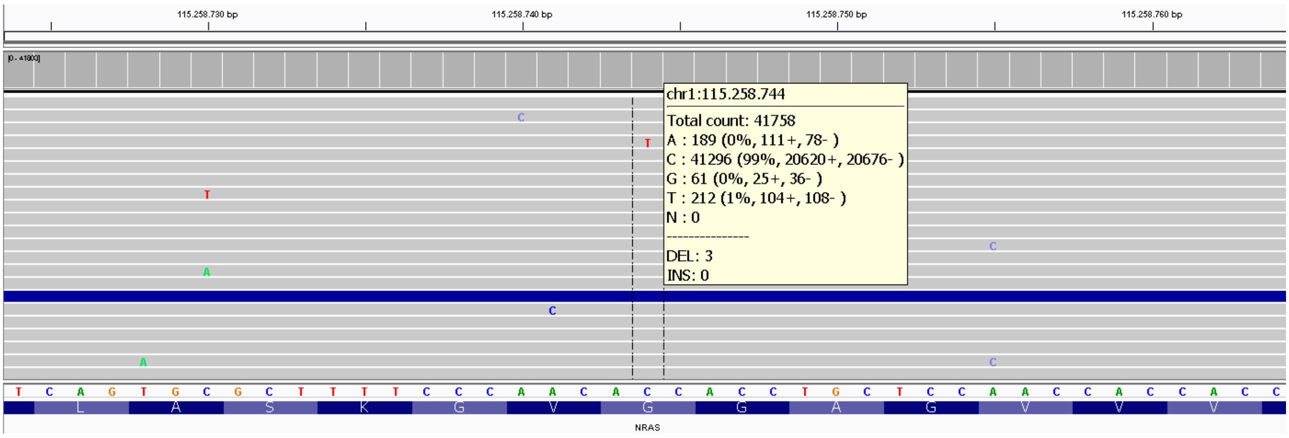

BRAF: NM_004333.5: c.1799T>A; p.(V600E)

P3

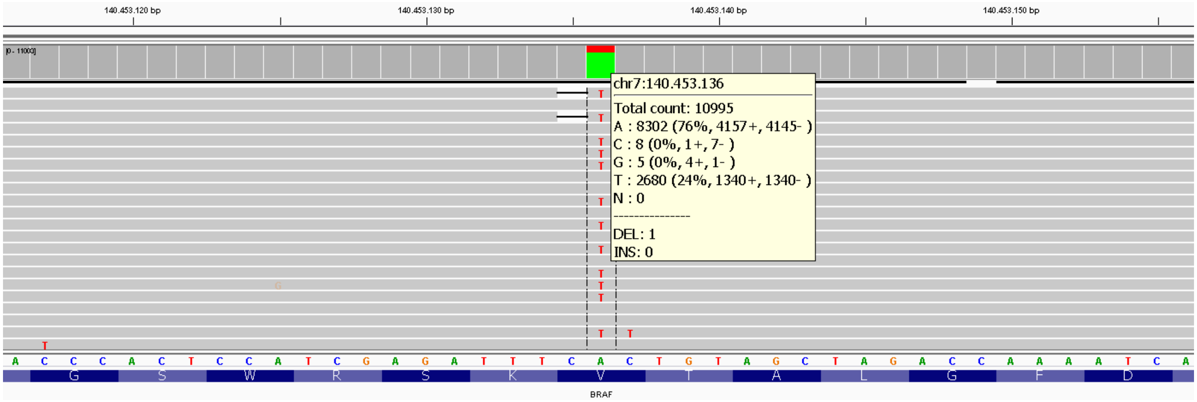

P39

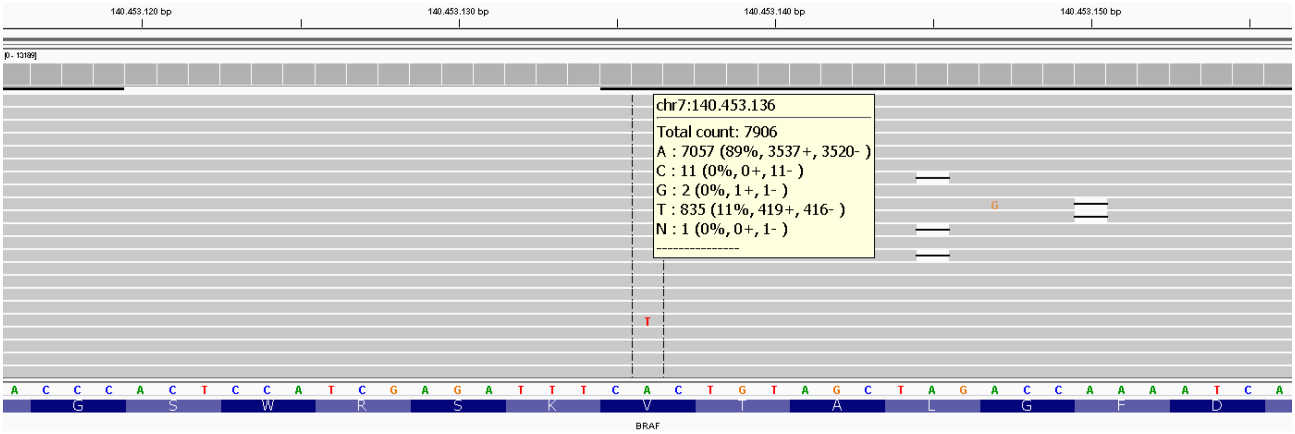

P55

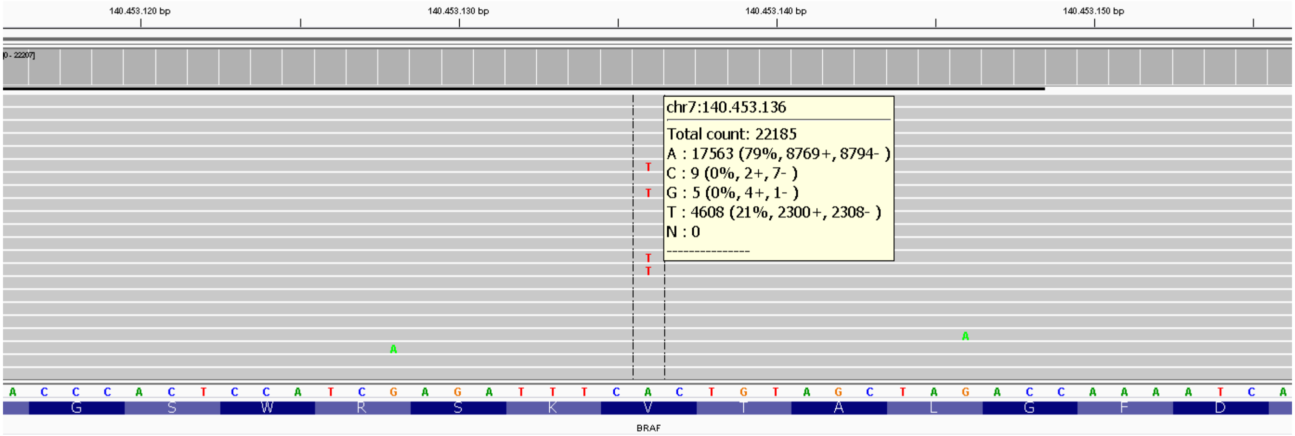

P64

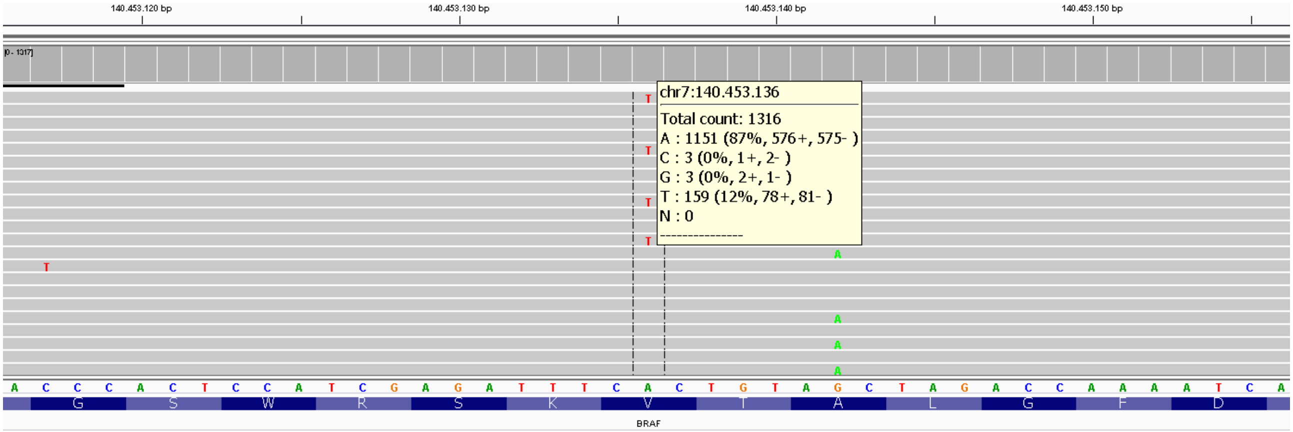

PIK3CA: NM_006218.3: c.1633G>A; p.(E545K) (P63)

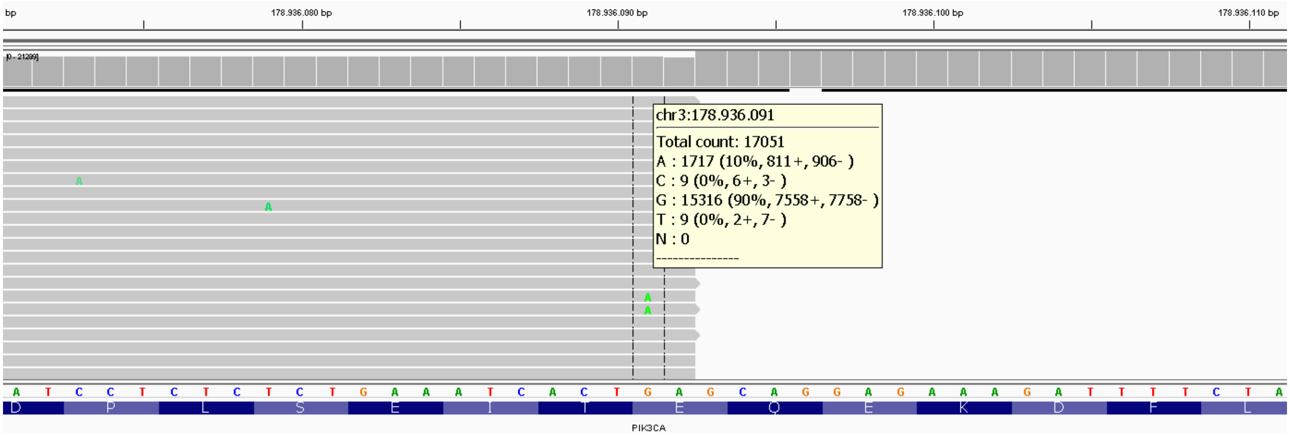

b) Sensitive patients

KRAS: NM_004985.4: c.437C>T; p.(A146V) (P28)

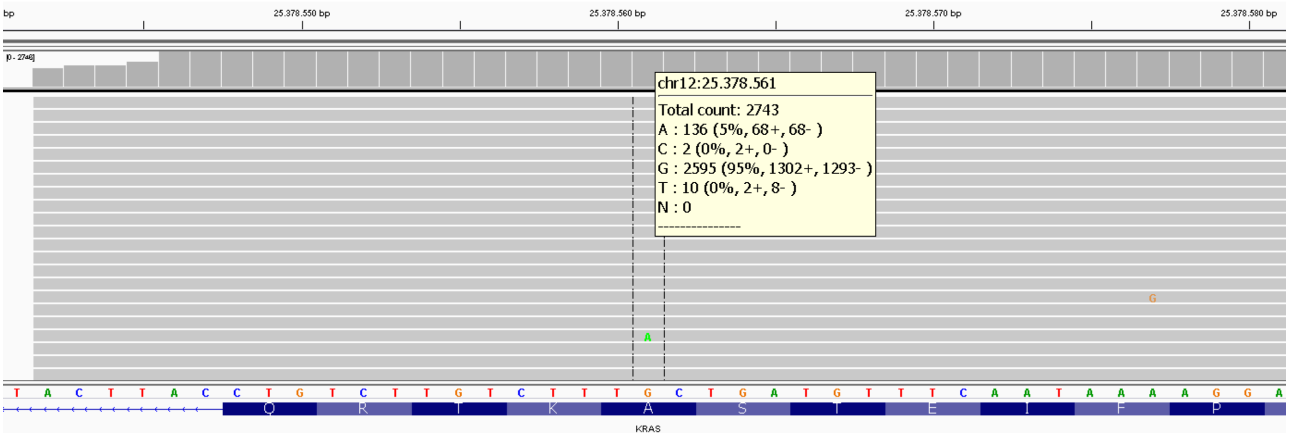

BRAF: NM_004333.5: c.1780G>A; p.(D594N) (P45)

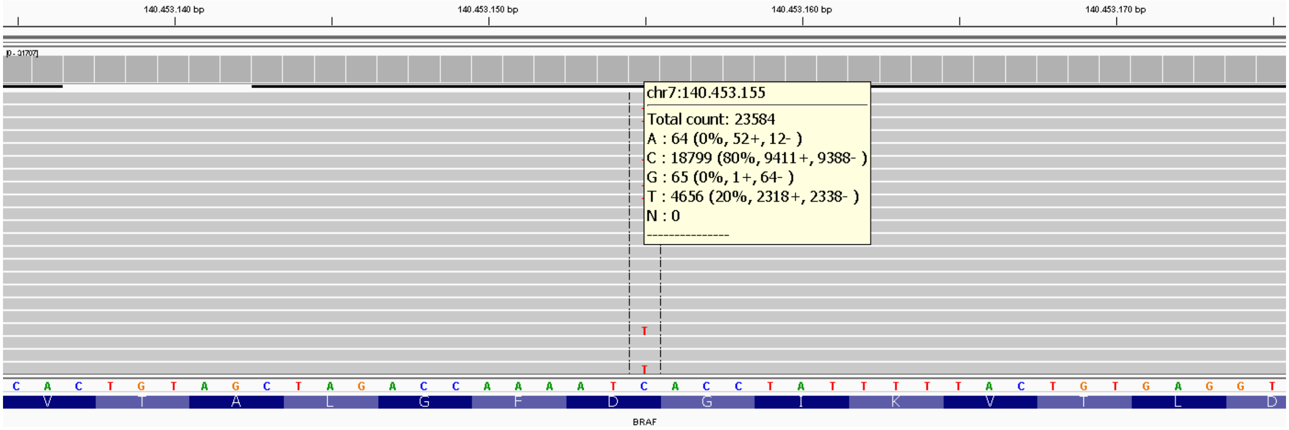

BRAF: NM_004333.5: c.1397G>C; p.(G466A) (P59)

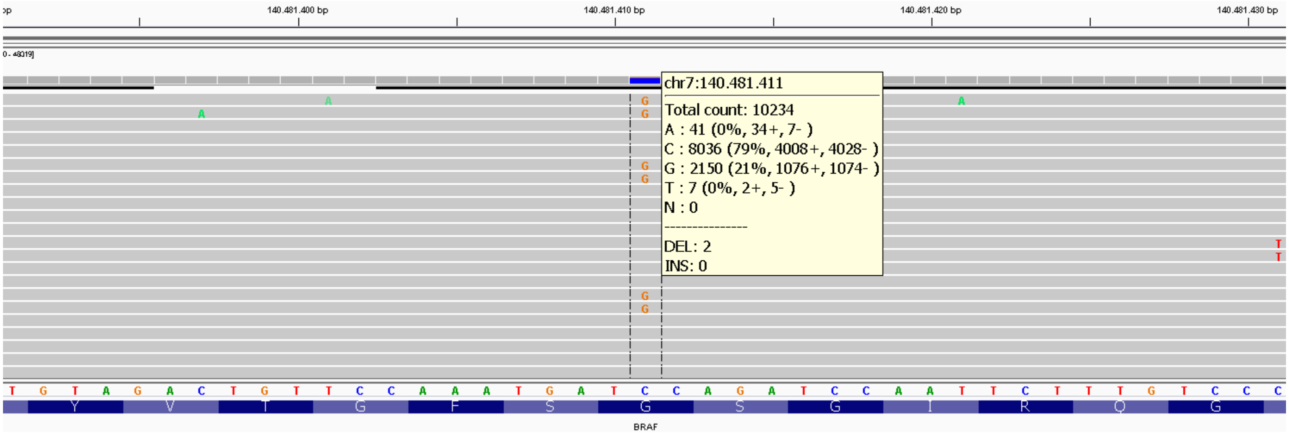

PIK3CA: NM_006218.3: c.1633G>A; p.(E545K) (P66)

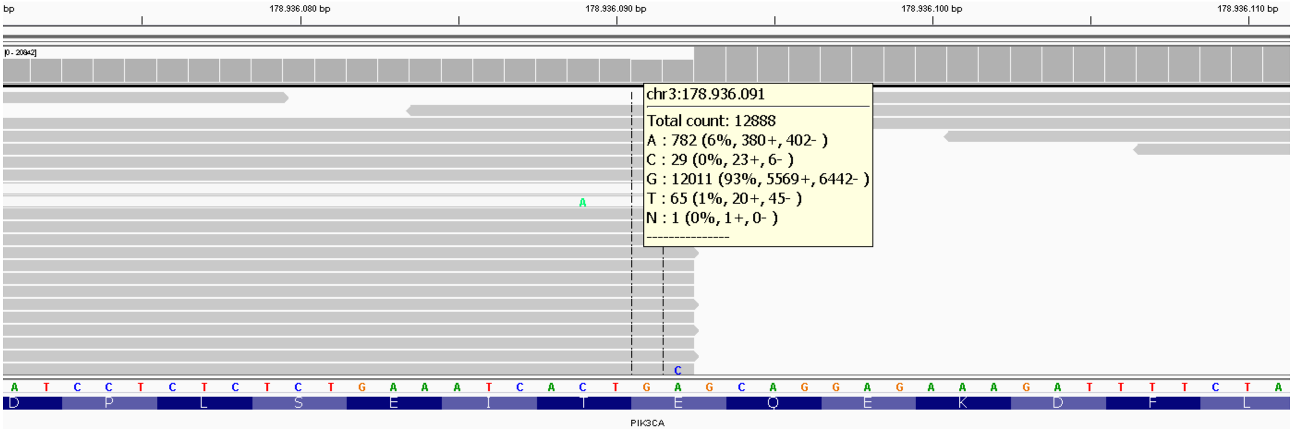

Supplementary figure S2.

IGF1R: NM_001291858.1: c.2003T>A; p.(I668N)

P4

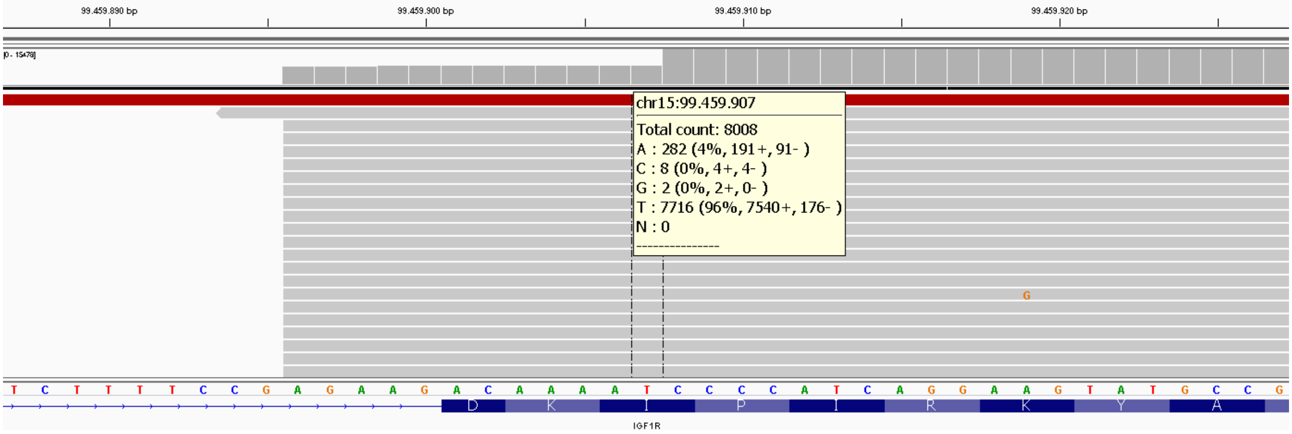

P9

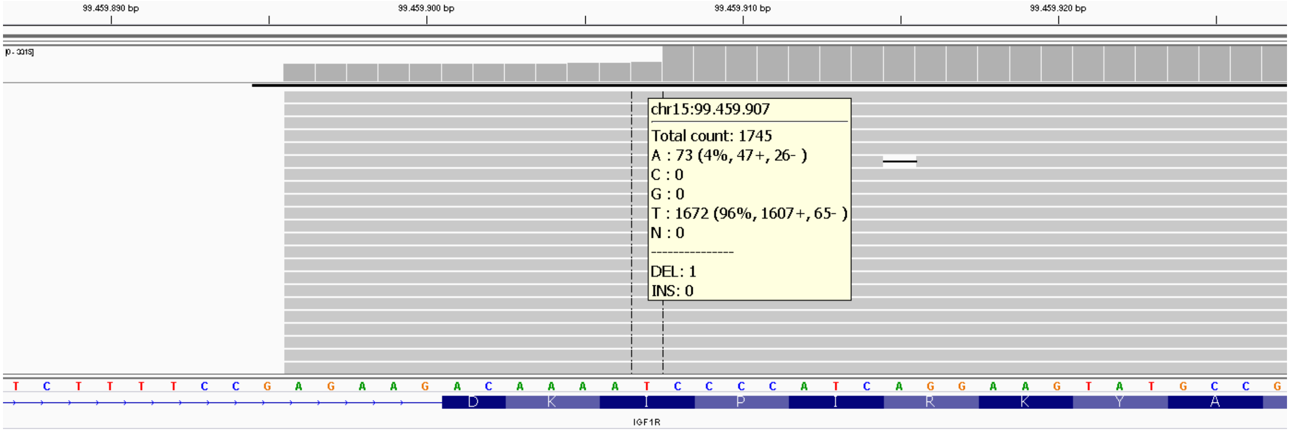

P10

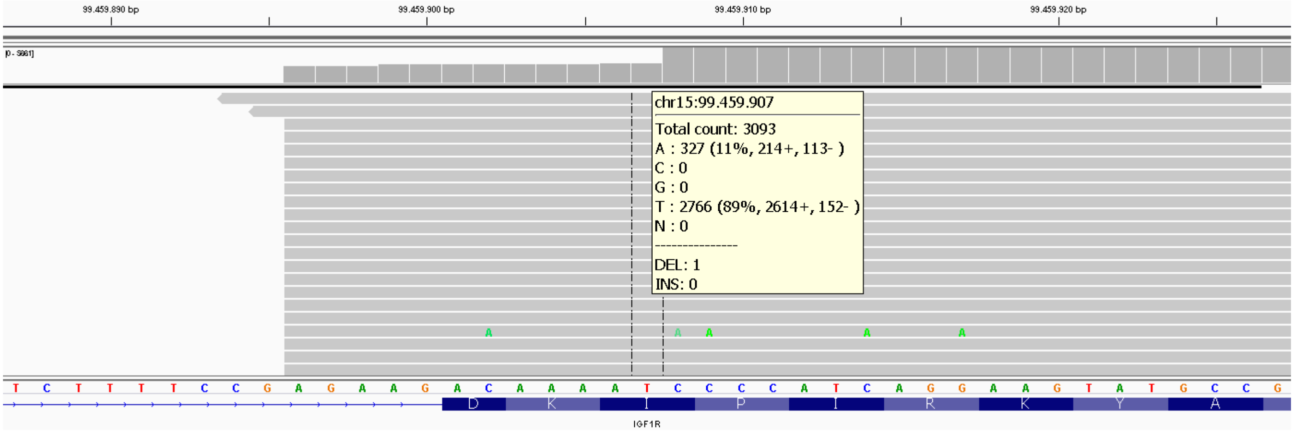

IGF1R: NM_001291858.1: c.3652G>A; p.(E1218K)

P1

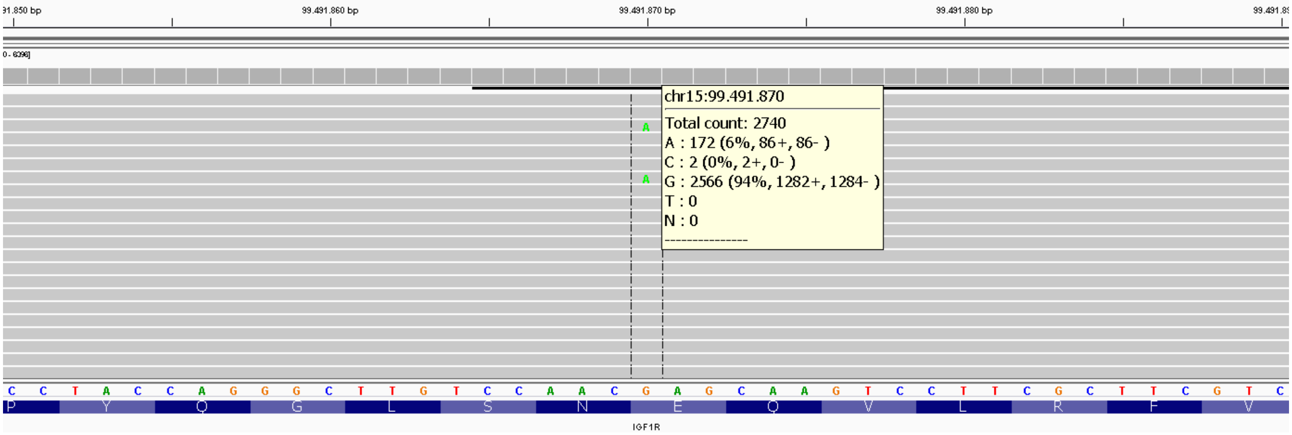

P2

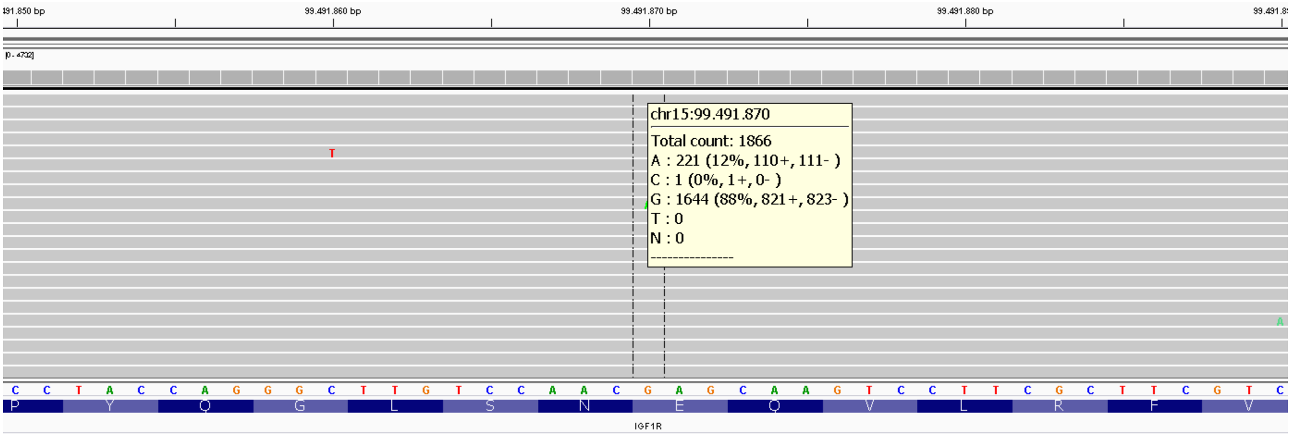

P9

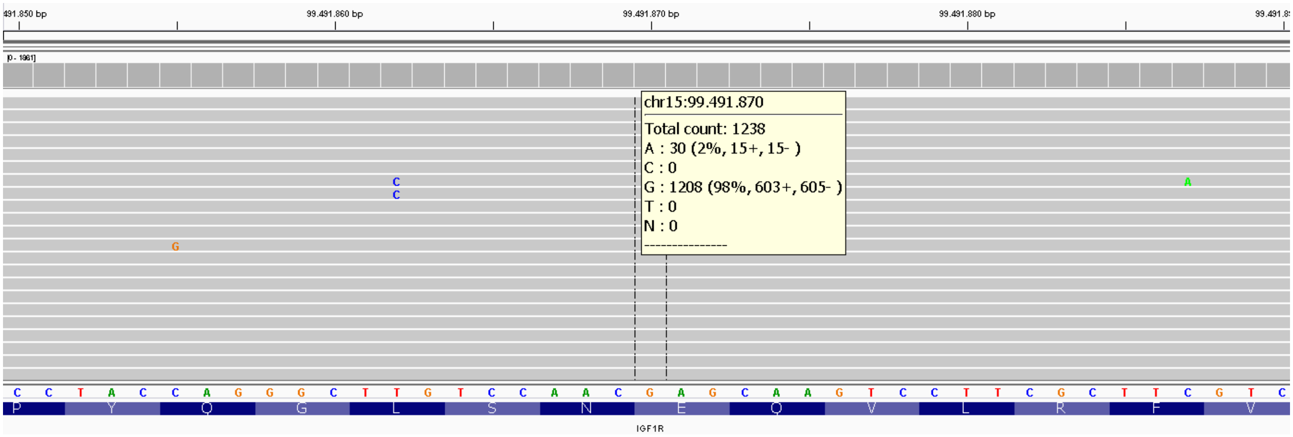

P61

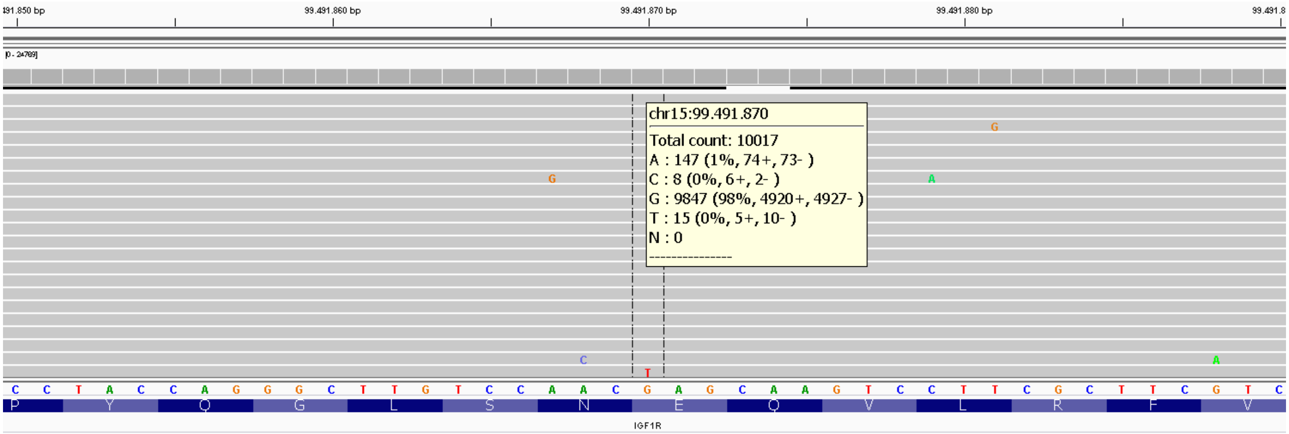

IRS2: NM_003749.2: c.3467C>T; p.(T1156M)

P12

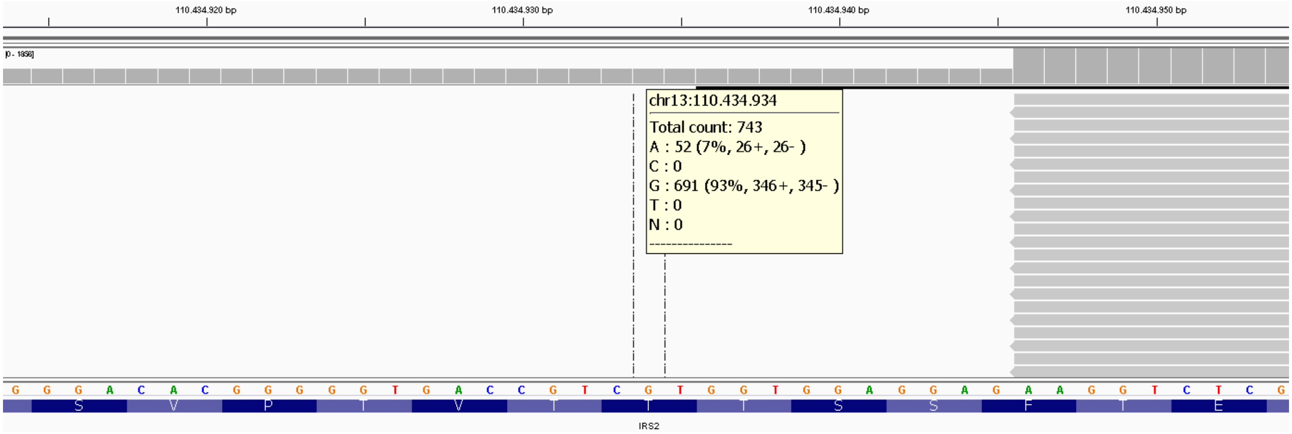

P14

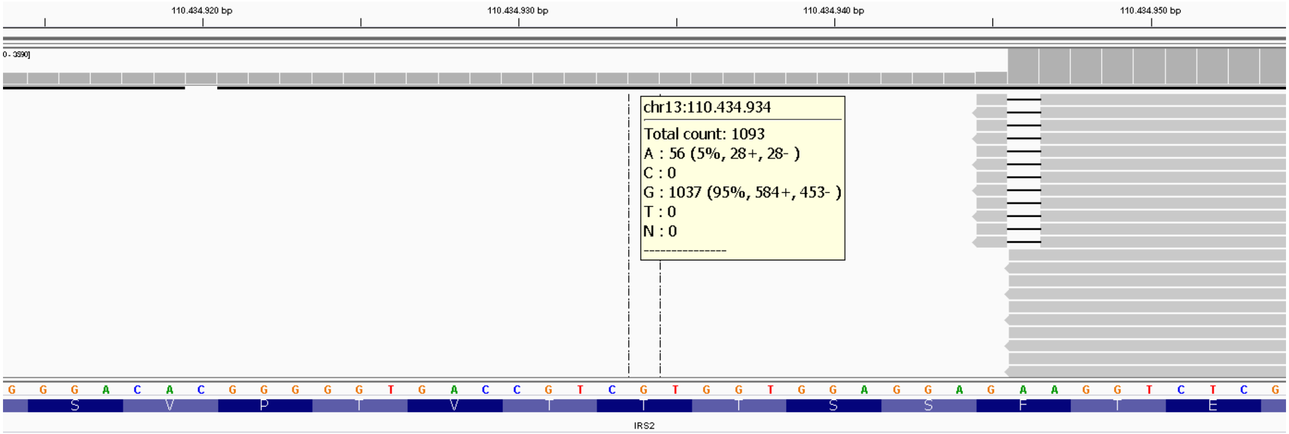

P57

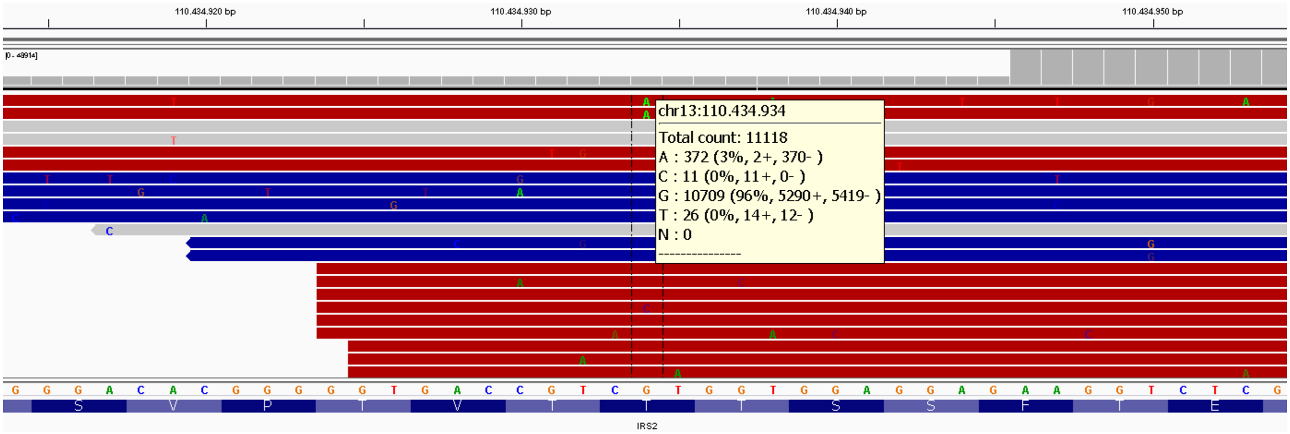

LRIG1: NM_015541: c.456G>A; p.(T152T)

P12

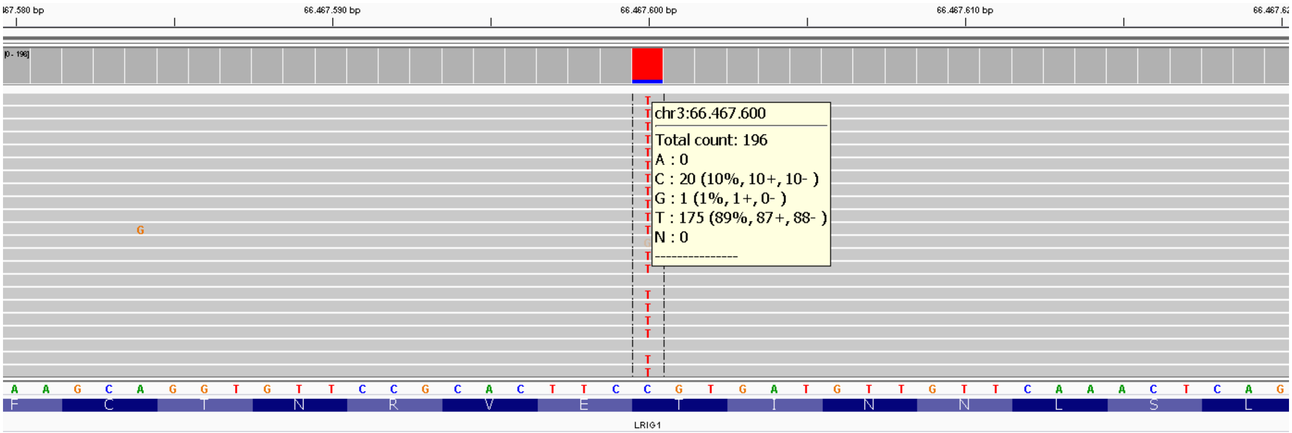
66.467.590 bp
66.467.600 bp
66.467.610 bp
chr3:66.467.600
Total count: 196
A : 0
C : 20 (10%, 10+, 10-)
G : 1 (1%, 1+, 0-)
T : 175 (89%, 87+, 88-)
N : 0
LRIG1

P57

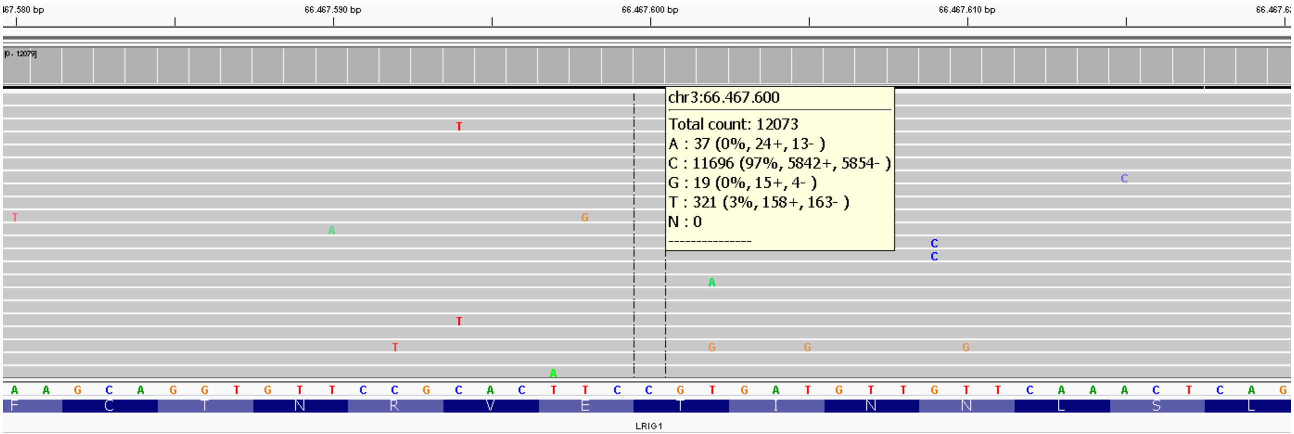
66.467.590 bp
66.467.600 bp
66.467.610 bp
chr3:66.467.600
Total count: 12073
A : 37 (0%, 24+, 13-)
C : 11696 (97%, 5842+, 5854-)
G : 19 (0%, 15+, 4-)
T : 321 (3%, 158+, 163-)
N : 0
LRIG1

P63

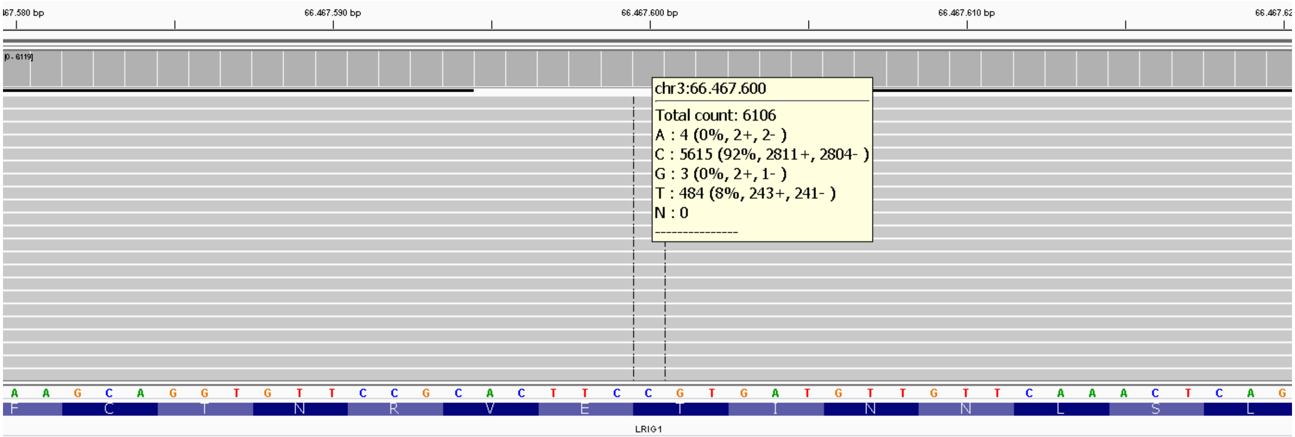
66.467.590 bp
66.467.600 bp
66.467.610 bp
chr3:66.467.600
Total count: 6106
A : 4 (0%, 2+, 2-)
C : 5615 (92%, 2811+, 2804-)
G : 3 (0%, 2+, 1-)
T : 484 (8%, 243+, 241-)
N : 0
LRIG1

P67

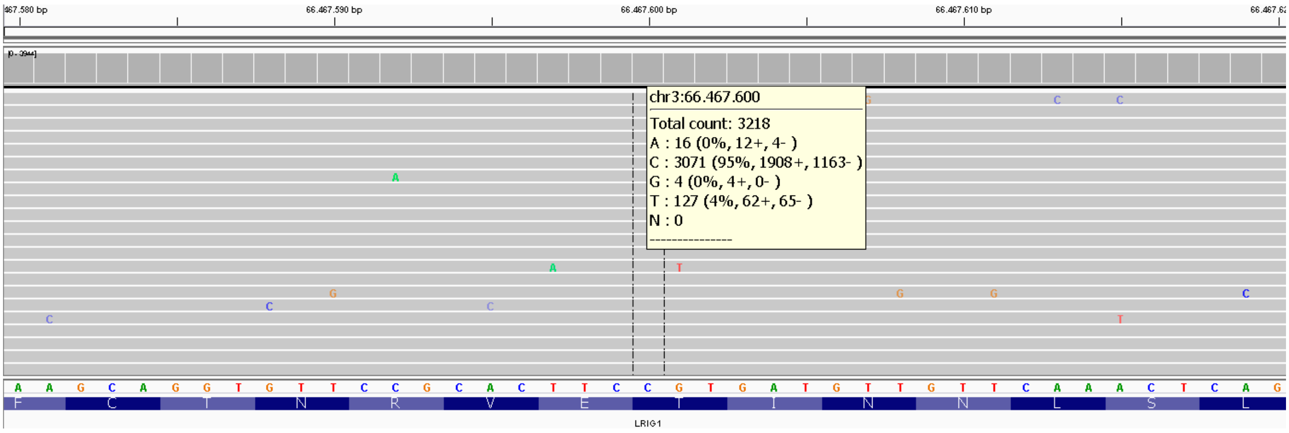

LRIG2: NM_014813.2: c.2090C>T; p.(S697L)

P1

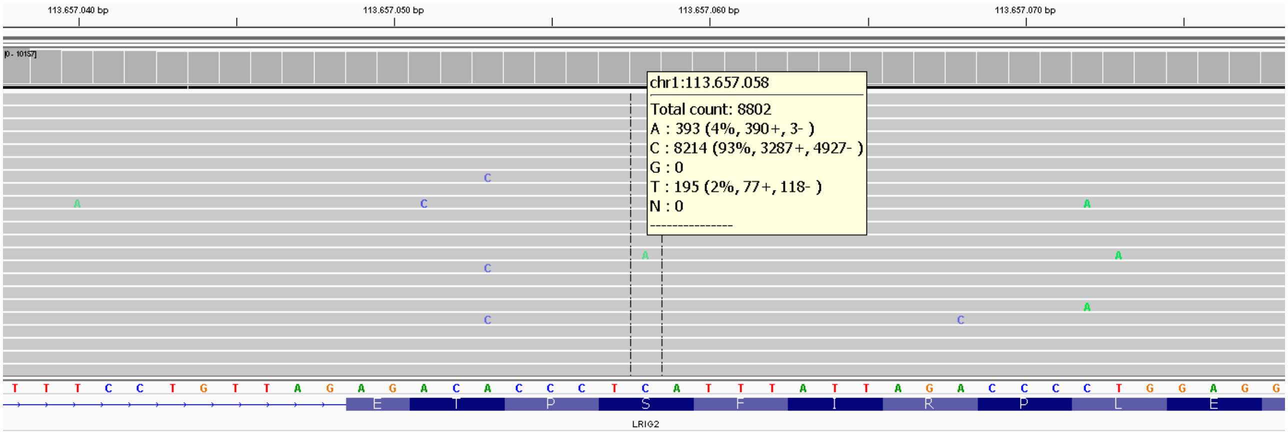

P10

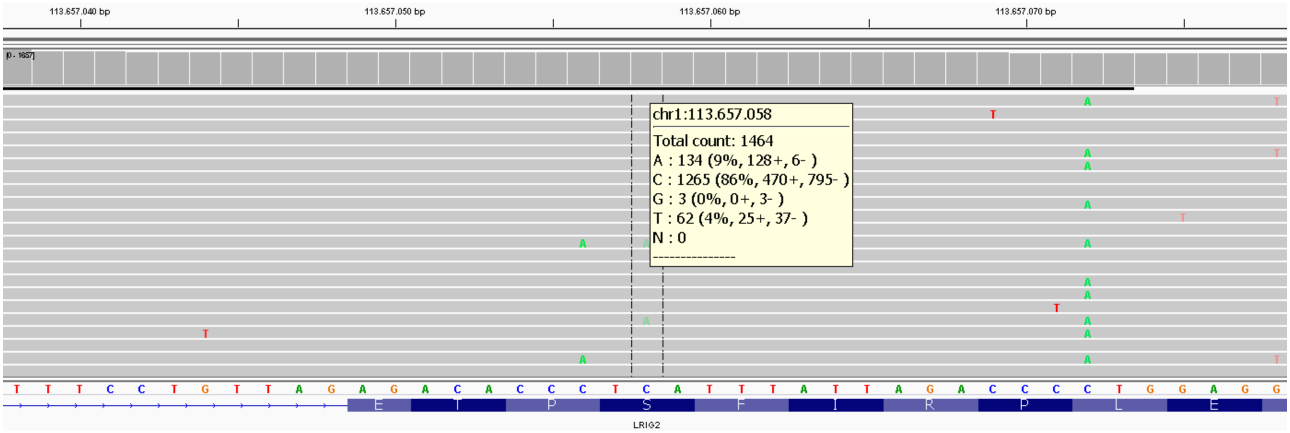

P12

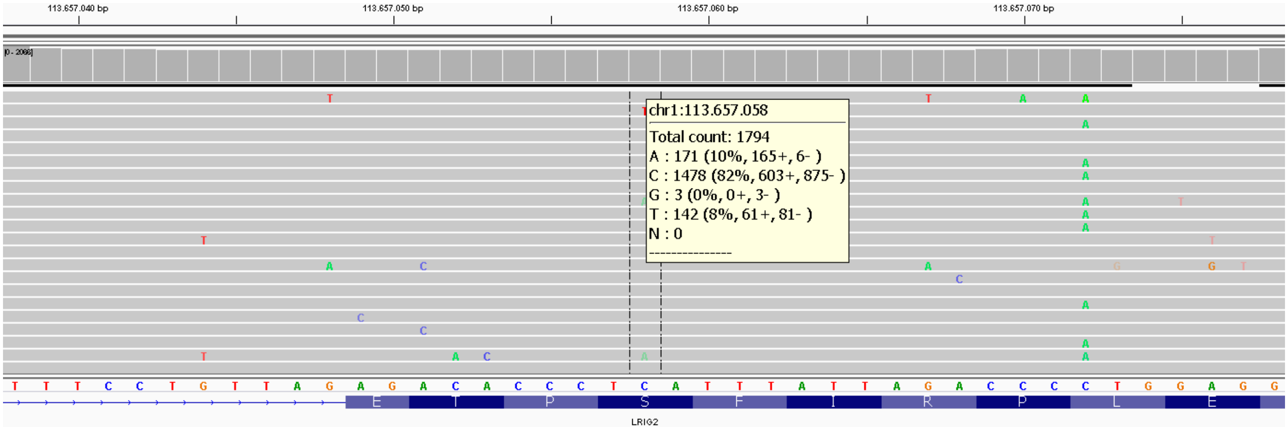

LRIG3: NM_001136051.2: c.2434G>A; p.(V812M)

P1

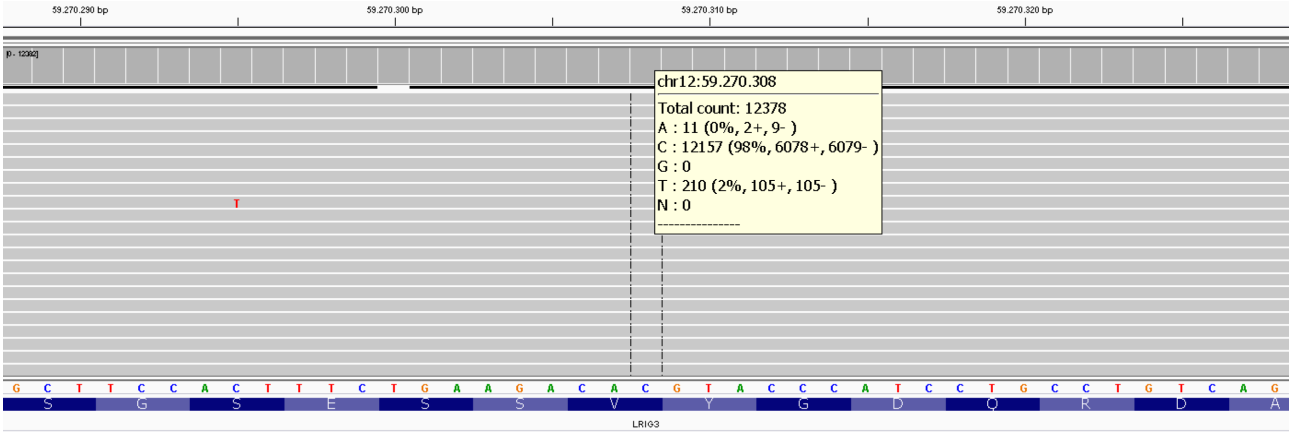

P2

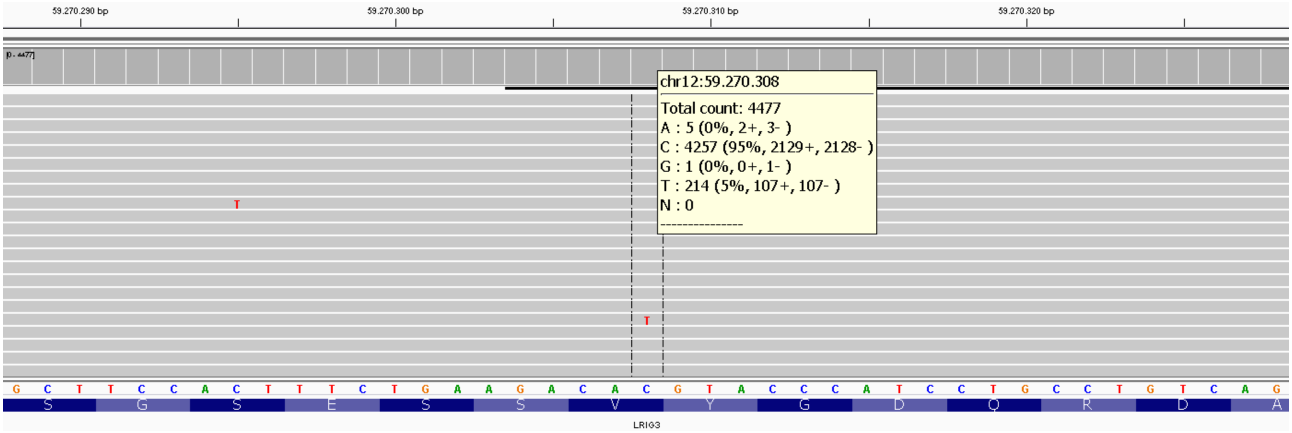

P9

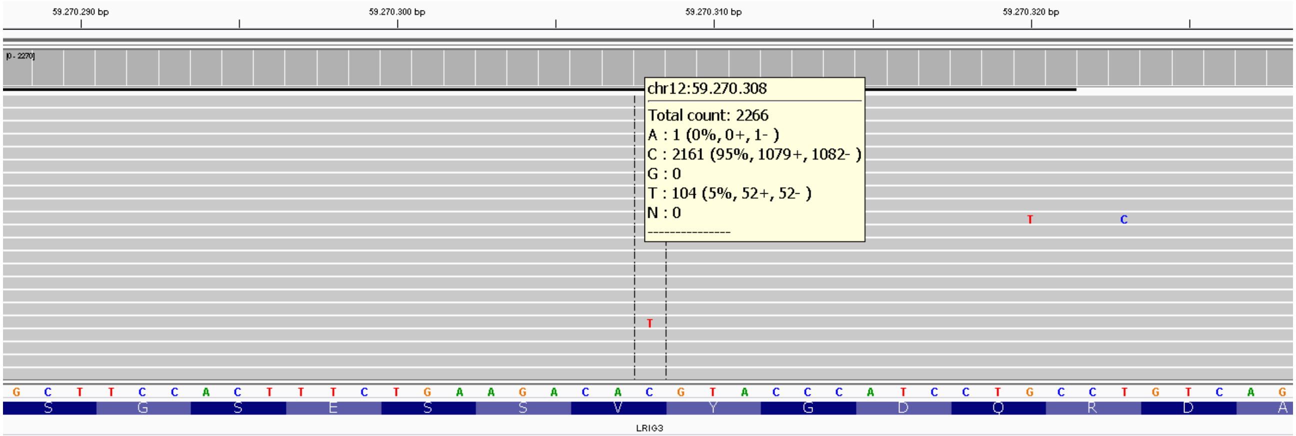

*NRAS: NM_002524.3: c.344del; p.(G115Efs*46)*

P9

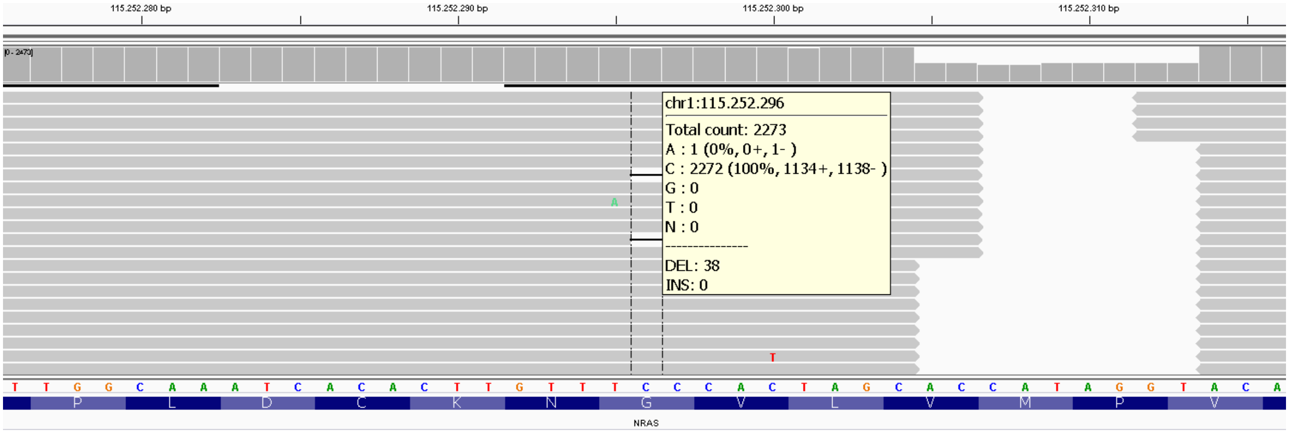

P10

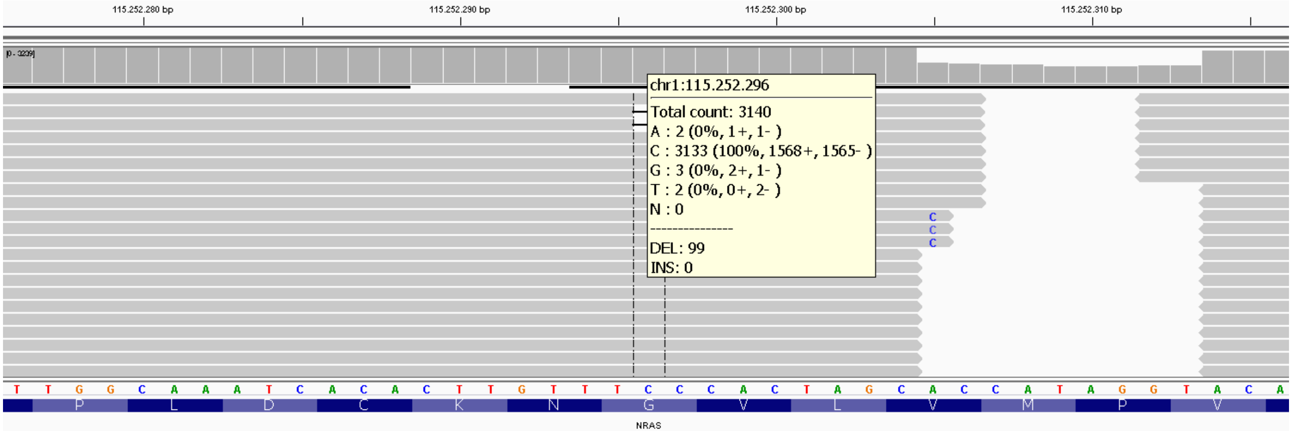

P63

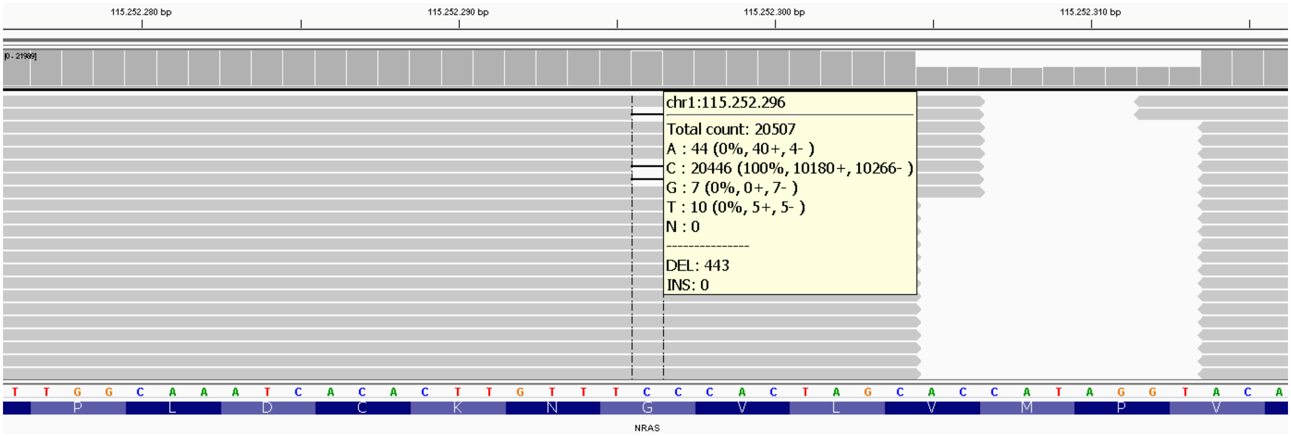

PDGFRA: NM_001347828: c.903G>A; p.T301T

P52

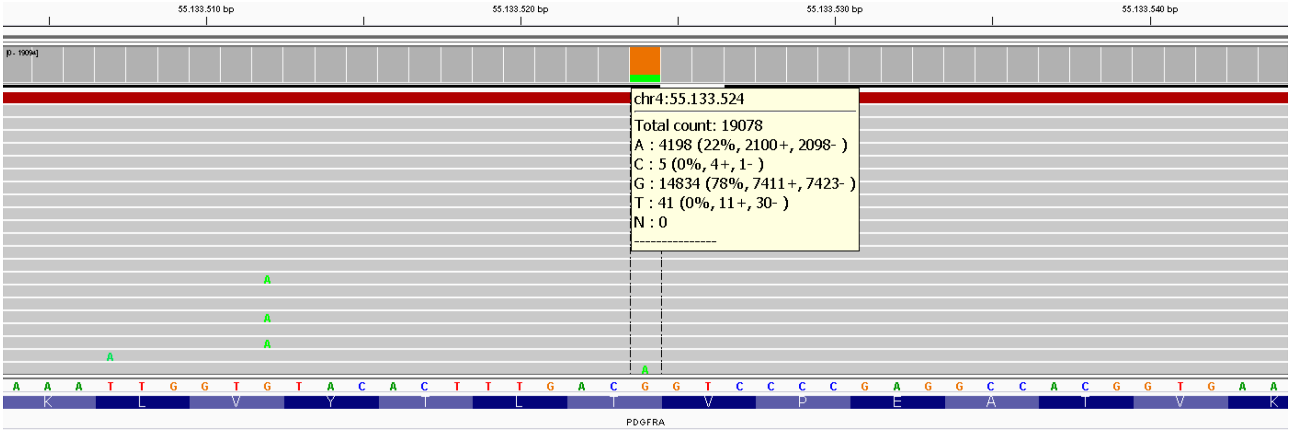

P63

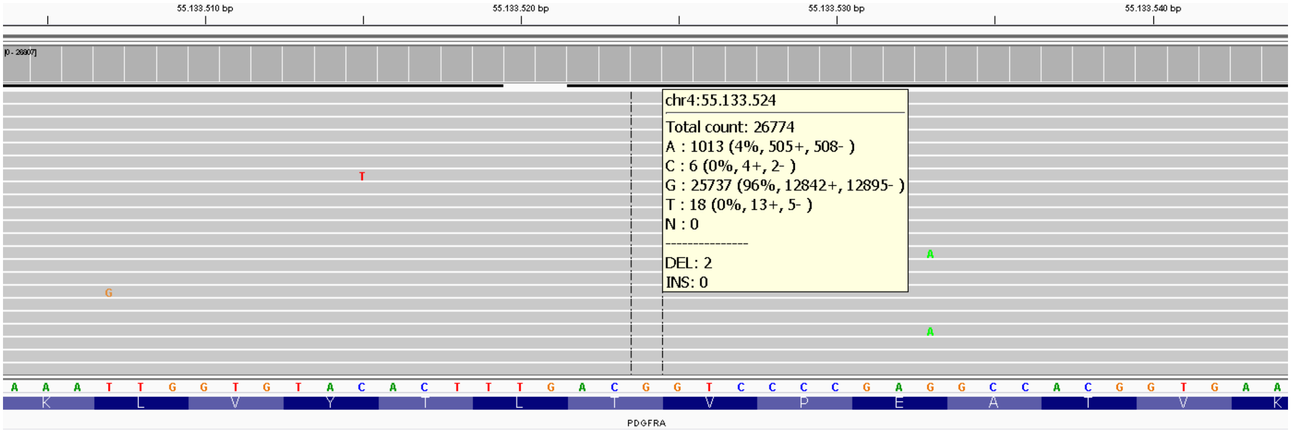

P67

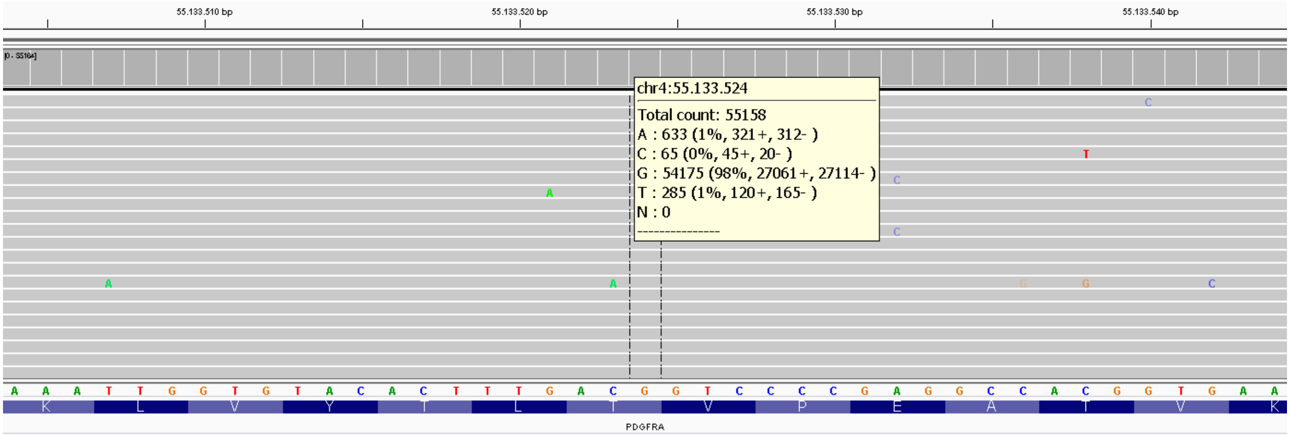
55.133.510 bp
55.133.520 bp
55.133.530 bp
55.133.540 bp
chr4:55.133.524
Total count: 55158
A : 633 (1%, 321+, 312-)
C : 65 (0%, 45+, 20-)
G : 54175 (98%, 27061+, 27114-)
T : 285 (1%, 120+, 165-)
N : 0
PDGFRA
